# Exploring the mediating factors linking adverse childhood experiences to traditional Chinese quality of life among older adults: a multi-factorial analysis

**DOI:** 10.3389/fpubh.2025.1598440

**Published:** 2025-09-16

**Authors:** Mingjun Sun, Na Zhang, Yuxin Zhang, Wurong Chang, Haolin Cao, Qiang Zhou, Limin Li, Zijian Tang, Yulu Zhou, Huiwen Li, Yisong Yao, Junxiao Si

**Affiliations:** ^1^Key Laboratory of Digital-Intelligent Disease Surveillance and Health Governance, North Sichuan Medical College, Nanchong, Sichuan, China; ^2^School of Nursing, Health Science Center, Xi’an Jiaotong University, Xi’an, China; ^3^Nanchong Hospital of Traditional Chinese Medicine, Nanchong, Sichuan, China; ^4^Guangxi Medical University, Nanning, Guangxi, China; ^5^Qingdao University, Qingdao, Shandong, China

**Keywords:** adverse childhood experiences, traditional Chinese medicine, quality of life, perceived social support, older adults, chronic diseases

## Abstract

**Purpose:**

The global population is aging rapidly, making the facilitation of positive aging a pivotal issue that necessitates immediate global attention. This study investigates the association between adverse childhood experiences (ACEs) and the Chinese medicine quality of life (CMQL) in older adults, guided by life course theory and a traditional Chinese medicine perspective.

**Methods:**

A total of 1,119 older adults individuals were included in this study. Logistic regression and propensity score matching (PSM) were used to explore the relationship between ACEs and CMQL. Structural equation modeling was employed to analyze the mediating roles of balance constitution, chronic disease, and perceived social support in the relationship between ACEs and CMQL.

**Results:**

There was a significant negative association between ACEs and CMQL among older adults. Individuals with ACEs displayed compromised balance constitution, a higher prevalence of chronic diseases, and a lower level of CMQL. High levels of perceived social support were identified as a protective factor, effectively mitigating the adverse effects of ACEs on CMQL among older adults individuals.

**Conclusion:**

While the study uncovered a significant negative correlation between ACEs and CMQL in older adults, it is important to acknowledge the limitations of drawing causal relationships from cross-sectional data. Moreover, the findings underscore the protective role of perceived social support in mitigating the detrimental impacts of ACEs on CMQL in the older adults population. Therefore, prioritizing interventions that bolster social support networks may offer significant benefits in enhancing the overall quality of life for older adults.

## 1 Introduction

The global population of older adults is rapidly expanding (1). In 2023, it was estimated that there were 961 million older adults worldwide (2). In China, the older adults population has surged to 310 million, accounting for 22.0% of the total population (1, 2). Projections suggest that by 2030, China’s population older adults 60 and above will surpass 400 million (3). The increasing number of older adults in China has brought healthy aging into sharp focus across all sectors of society (4). Studies have revealed that more than 80% of older adults suffer from at least one non-communicable chronic disease, highlighting the prevalence of suboptimal healthy conditions in this demographic (3). In response to the paradigm shift toward promoting healthy aging, there is a growing emphasis on assessing the health-related quality of life of older adults, necessitating an evaluation of their current status and the factors influencing it (1–4).

Health-related quality of life is subjective and encompasses an individual’s physical, cognitive, emotional, and social participation or support system (5). Previous research in China have predominantly utilized tools like the EuroQol Five Dimensions Questionnaire (EQ-5D) and the short-form 6 dimensions (SF-6D) to measure health-related quality of life (6–8). However, since health-related quality of life is culturally bound, evaluation metrics developed in Western contexts may not fully capture the Chinese perspective on health. The unique cultural and economic circumstances of older adults in China could make foreign scales partially or entirely unsuitable. For instance, a comparative study examined various health-related quality of life instruments from China and other countries (9). In these assessments, elements specific to Chinese instruments, such as emotional regulation, adaptation to weather, and social integration, were deemed significant by participants but were absent from widely used Western health-related quality of life tools (10).

In the realm of health concept in Chinese traditional culture, traditional Chinese medicine is always regarded as a system that effectively embodies the Chinese cultural perspective on health. Taking into account the unique health characteristics of the Chinese populace and drawing from Traditional Chinese Medicine (TCM) health theory, and perspectives, Zhou et al. introduced the Chinese Medicine Quality of Life Evaluation scale (CQ-11D), which offers a potentially more appropriate means of gauging quality of life among general Chinese population (11). This scale incorporates elements of TCM health theory that are absent in conventional Western health-related quality of life tools, such as appetite, stool, and dizziness. Moreover, it provides an effective method for conducting clinical and economic evaluations within the realm of TCM (12). Notably, it is widely considered that the EQ-5D is not sensitive enough for assessing sub-health conditions, and the SF-6D is not adequate for discriminating mild diseases (13, 14). The CQ-11D, applied to the Chinese population, has demonstrated a capacity to circumvent these issues (12). Therefore, this study employs the CQ-11D as a comprehensive instrument for surveying of the target population, aiming to provide an accurate depiction of Chinese medicine quality of life (CMQL).

Adverse childhood experiences (ACEs) encompass a range of potentially traumatic events that occur during an individual’s childhood, mainly including deprivation or threat-related events including physical, emotional, and social relationships (15). In accordance with life course theory, childhood stands out as a pivotal phase of development; with adverse events incidents during this period exerting a profound influence on one’s health trajectory (16). Exposure to ACEs has been linked to the onset of chronic diseases and comorbidity (15). Previous studies has also underscored the lasting neurological repercussions of ACEs well into adulthood, impacting facets such as social connection, depressive symptoms, and even cognitive impairment (3, 17). Additionally, Tabatabaei et al. have shed light on how the detrimental effects of ACEs can manifest in health-related quality of life assessments during later stages of life (18).

Perceived social support pertains to individuals’ subjective perceptions of assistance from friends, family, and significant others, alongside their psychological well-being. Prior studies have underscored its pivotal role in bolstering the physical and mental wellness of older adults (19, 20). Chronic diseases have also been linked to declines in health among the older adults (21). Besides, the theory of traditional Chinese medicine constitution (TCMC) represents a branch of TCM emphasizing that accentuates innate differences stemming from the parental genetics, while acquired disparities arise from factors like environments conditions and lifestyle behaviors (22). A series of studies have confirmed the correlation between TCMC, particularly the balance constitution (BC) and diverse chronic diseases (23, 24).

Building upon the evidence discussed earlier, we undertook an assessment of the present state of CMQL among older adults in Sichuan Province. Additionally, we delved into the relationship between ACEs and CMQL. We hypothesized that ACEs would be negatively associated with CQ-11D. And we investigated how chronic diseases, perceived social support and BC mediate this relationship among older adults. Our study specifically emphasizes the significance of life course theory in understanding these dynamics.

## 2 Materials and methods

### 2.1 Study design

From June to August 2024, we utilized a random number table method to select eight districts (counties) in Sichuan Province and employed quota sampling based on gender and regional population distributions. Following the Kendall sample size estimation method, we determined that a sample size of 1,068 older individuals was needed, which was increased by 20% to accommodate potential invalid questionnaires, resulting in a final inclusion of 1,119 valid questionnaires. Inclusion criteria for this study entailed: individuals older adults 60 years and above; capability to complete the survey; permanent residents in Sichuan Province; provision of informed consent and voluntary participation; and complete filling of the study material. Exclusion criteria encompassed: logical inconsistencies and missing key variables.

### 2.2 Measurement

#### 2.2.1 Covariate variables

Sex, education level, urban–rural distribution, and nationality were included as covariate variables based on the previous studies (3).

#### 2.2.2 Chinese medicine quality of life assessment scale (CQ-11D)

The primary outcome was Chinese Medicine Quality of Life (CQ-11D). This scale was compiled by Zhu et al. and endorsed through the industry standards of the China Association of Traditional Chinese Medicine. It contains 11 items: XD (movement and self-care), SY (appetite), DB (stool), SM (quality of sleep), JS (spirit, including being alive, energetic, and focused), TY (dizziness, including feeling dizzy in the mind, with eyes closed for minor cases, or spinning in front of the scene in serious cases, inability to stand), XH (palpitations, or feeling restless), TT (pain), PL (fatigue), FZ (irritability), JL (anxiety, worried, anxious, nervous, restless), These components are categorized into two dimensions: “Body and Spirit Together – Body” and “Body and Spirit Together – Spirit.” Each item response includes four levels: “very good” (four points), “relatively good” (three points), “relatively poor” (two points) and “very poor” (one point). The health utility value is calculated using the “CQ-11D health utility value system” as an evaluation index for quality of life. The formula for calculation is: 1–0.083 × XD2–0.355 × XD3–0.500 × XD4–0 × SY2–0.102 × SY3, where a higher health utility value signifies an enhanced quality of life (25).

#### 2.2.3 The traditional Chinese medicine constitution classification and self-assessment scale (TCMCC)

The TCMCC was issued by the China Association of Traditional Chinese Medicine (26). It is formulated based on the principles of TCMC theory to identify an individual’s constitutional type. This assessment categorizes constitution into nine fundamental types, which include yang-deficiency constitution (YADC), qi-deficiency constitution (QDC), qi-stagnation constitution (QSC), phlegm-dampness constitution (PDC), yin-deficiency constitution (YIDC), damp-heat constitution (DHC), blood stasis constitution (BSC), balance constitution (BC), and inherited special constitution (ISC). Each item is evaluated based on the intensity of symptoms to ascertain the constitution type.

#### 2.2.4 Adverse childhood experiences questionnaire

This questionnaire was self-compiled by the research group based on the previous studies (15, 17, 27–29). It primarily explores 11 categories of ACEs, including: “parental death,” “parental physical/mental disability,” “parental long-term illness,” “poor parental relationships,” “hunger,” “poor parental mental health,” “parents lacking formal education,” “parental divorce,” “frequent abuse (verbal, physical),” “forced school dropout” and “parents being indifferent to you.”

#### 2.2.5 Social support rating scale (SSRS)

The Social Support Rating Scale (SSRS) was compiled by Xiao et al. in 1986 and later revised in 1990, used to measure individual’s social support status. This self-report scale comprises 10 items, with a total score of 8 to 44 points. It includes three dimensions: subjective support (items 1 and 3–5, scoring between 4 and 16 points), objective support (items 2, 6, and 7, scoring between 4 and 16 points), and the utilization of social support (items 8–10, scoring between 3 and 12 points). Higher scores within each dimension indicate a greater degree of social support. Scores below 20 suggest low support, scores between 20 and 30 indicate average support, and scores ranging from 31 to 40 indicate satisfactory support (30).

### 2.3 Data analysis

The data were analyzed using SPSS 27.0, Stata 16.0, and AMOS 26.0. Initially, the suitability of the data was assessed using the Kaiser-Meyer-Olkin (KMO) measure and Bartlett’s test of sphericity, followed by principal component analysis. Reliability and validity tests were conducted to demonstrate that strong convergent and composite validity of the scales employed in this study.

Secondly, descriptive statistical analyses were performed to compare variable scores among older adults with different demographic characteristics. Spearman correlation analysis was then used to explore the relationships among the key variables. Multiple linear regression was employed to investigate the correlation between ACEs and CMQL. To mitigate selection bias, propensity score matching (PSM) was implemented, aligning the distribution of covariates in the treated and control groups. Five PSM methods were adopted, including one-to-one matching, one-to-four matching, radius matching, and kernel matching to ensure the similarity of treatment and control groups based on their propensity scores and control variables. Finally, mediating analysis was performed to elucidate the pathway linking ACEs and CMQL. All statistical tests in this study were performed at a significance level of *p* < 0.05 via a two-tailed approach.

### 2.4 Applicability test

Using SPSS 27.0, principal component analysis was performed on five variables to reduce the dimensionality of the dataset, accompanied by suitability testing. The outcomes are detailed in Supplementary Table 1. The analysis yielded a KMO test value of 0.931 was obtained, and Bartlett’s spherical test chi-square statistic indicated a significance level of <0.001, affirming that principal component analysis satisfactorily met the feasibility criteria.

### 2.5 Principal component analysis

Principal component analysis was conducted on the variables within the measurement questionnaire using SPSS 27.0. As depicted in Supplementary Table 2, four principal components were extracted, with a cumulative variance contribution rate of 61.623%. This suggested that these principal components effectively capture the essence of the original data. The variance accounted for by the first principal component was 25.584%, which fell below the critical threshold of 40%, indicating that the homogeneity criterion was within an acceptable range, thus meeting the prerequisites for further data analysis.

### 2.6 Reliability and validity test

As shown in Supplementary Table 3, Cronbach’s coefficient of reliability test was used to analyze the internal consistency of each dimension. The results revealed that the Cronbach’s coefficients of the scales used in this study range from 0.821 to 0.861, demonstrating robust internal consistency. Factor analysis was conducted to confirm the convergent validity of the measurement model, examining the average variance extracted (AVE) and composite reliability (CR) of the scale dimensions. The results showed that the standardized loading range of CQ-11D items was 0.482–0.762, with AVE = 0.414 < 0.5, but CR = 0.884, greater than 0.6, indicating that the convergent validity was acceptable. The standardized loading range of the TCMCC and SSRS items was 0.679–0.835, all above 0.5, with AVE ranging from 0.578 to 0.585, both above 0.5, and CR ranging from 0.809 to 0.925, all above 0.7, indicating commendable convergent validity and strong composite reliability of the scale dimensions.

## 3 Results

### 3.1 Descriptive statistics

A total of 1,119 older adults were included in this study, 77.03% of whom were older adults between 60 and 75. Table 1 illustrated the variable assignments and fundamental characteristics of the study population. 53.08% were female, and 81.68% reported at least one ACE. The median score of SSRS among the surveyed individuals was 41.00 (IQR 36.00–45.00), while the median score for CQ-11D was 0.91 (IQR 0.79–0.95).

**Table 1:** Variables and basic characteristics.

Category	*N* (%)	Variables [Score, M (IQR)]	
		CQ-11D	SSRS
*N*	1,119	0.91(0.79, 0.95)	41.00(36.00, 45.00)
Age			
60 ~ 65	330(29.49%)	0.92(0.85, 0.97)	42.00(36.75, 46.00)
66 ~ 70	286(25.56%)	0.92(0.79, 0.93)	41.00(36.75, 46.00)
71 ~ 75	246(22.00%)	0.89(0.79, 0.95)	40.00(34.00, 46.00)
76 ~ 80	142(12.69%)	0.85(0.71, 0.93)	40.00(34.75, 43.25)
81 ~ 85	72(6.43%)	0.83(0.60, 0.93)	40.00(32.00, 44.75)
86 ~ 90	31(2.77%)	0.82(0.60, 0.93)	38.00(31.00, 42.00)
≥91	12(1.07%)	0.74(0.32, 0.85)	38.50(30.25, 40.75)
*H*		78.510	23.566
*P*		<0.001	<0.001
Sex			
Male	525(46.92%)	0.92(0.81, 0.96)	40.00(35.00, 44.00)
Female	594(53.08%)	0.88(0.77, 0.94)	41.00(36.00, 46.00)
*Z*		-2.912	-2.140
*P*		0.004	0.032
Urban-country distribution			
Urban	615(54.96%)	0.90(0.79, 0.95)	40.00(35.00, 44.00)
Country	504(45.04%)	0.92(0.78, 0.95)	42.00(36.00, 46.00)
*Z*		−0.122	−3.931
*P*		0.903	<0.001
Nationality			
Han	1,079(96.43%)	0.91(0.79, 0.95)	41.00(36.00, 45.00)
Other	40(3.57%)	0.85(0.59, 0.96)	38.50(31.25, 43.00)
*Z*		−1.147	−2.103
*P*		0.252	0.035
Education level			
Illiterate	296(26.45%)	0.85(0.71, 0.93)	40.00(32.00, 44.00)
Primary and junior high school	602(53.80%)	0.90(0.79, 0.95)	40.00(36.00, 45.00)
Senior high school or vocational school	136(12.15%)	0.92(0.83, 0.96)	42.00(38.00, 45.75)
College and above	85(7.60%)	0.92(0.87, 0.96)	44.00(38.50, 48.00)
*H*		32.498	32.790
*P*		<0.001	<0.001
Chronic diseases			
Yes	914(81.68%)	0.97(0.92, 1.00)	43.00(37.00, 47.00)
No	205(18.32%)	0.87(0.76, 0.93)	40.00(35.00, 45.00)
*Z*		−12.361	−3.567
*P*		<0.001	<0.001
Adverse childhood experiences			
Yes	914(81.68%)	0.93(0.85, 0.97)	42.00(37.00, 47.00)
Parental death	200(17.87%)	0.85(0.70, 0.93)	39.00(33.00, 44.00)
Parental physical/mental disability	72(6.43%)	0.83(0.72, 0.91)	39.00(32.00, 44.00)
Parental long-term illness	120(10.72%)	0.83(0.64, 0.90)	40.00(36.00, 43.00)
Poor parental relationships	169(15.10%)	0.84(0.70, 0.92)	39.00(33.00, 42.00)
Hunger	670(59.87%)	0.87(0.77, 0.93)	40.00(35.00, 44.25)
Poor parental mental health	164(14.66%)	0.84(0.70, 0.92)	40.00(33.00, 44.00)
Parents lacking formal education	908(81.14%)	0.88(0.78, 0.94)	40.50(35.00, 45.00)
Frequent abuse	49(4.38%)	0.76(0.65, 0.88)	33.00(29.00, 41.00)
Forced school dropout	404(36.10%)	0.85(0.72, 0.93)	40.00(35.00, 44.00)
Parents being indifferent to you	149(13.32%)	0.84(0.66, 0.92)	39.00(31.50, 42.00)
Parental divorce	47(4.20%)	0.86(0.73, 0.93)	38.00(28.00, 42.00)
Other	39(3.49%)	0.86(0.77, 0.96)	39.00(33.00, 45.00)
No	205(18.32%)	0.88(0.77, 0.93)	40.00(35.00, 45.00)
*Z*		−6.760	−2.890
*P*		<0.001	0.004

Univariate ANOVA was conducted to analyze CQ-11D and SSRS scores among older adults with varying demographic characteristics. Significant unadjusted differences in CQ-11D were observed across sex (*Z* = −2.912, *p* = 0.004), education level (*Z* = 32.498, *p* < 0.001), chronic diseases (*Z* = −12.361, *p* < 0.001), and ACEs exposure (*Z* = −6.760, *p* < 0.001). Significant unadjusted differences in SSRS scores were observed across sex (*Z* = −2.140, *p* = 0.032), urban-country distribution (*Z* = −3.931, *p* < 0.001), nationality (*Z* = −2.103, *p* = 0.035), education level (*Z* = 32.790, *p* < 0.001), chronic diseases (*Z* = −3.567, *p* < 0.001), and ACEs exposure (*Z* = −2.890, *p* = 0.004). More details were shown in Table 1.

### 3.2 Correlation analysis

Pearson correlation analysis was conducted to assess the relationships among ACEs, chronic diseases, BC, SSRS, and CQ-11D (Supplementary Table 4). The findings revealed significant positive correlations between ACEs and chronic diseases (*r* = 0.248, *p* < 0.001), while significantly negatively correlations were observed between ACEs and BC (*r* = −0.138, *p* < 0.001), SSRS (*r* = −0.086, *p* < 0.01), and CQ-11D (*r* = −0.202, *p* < 0.001). Furthermore, chronic diseases exhibited significantly negative associations with BC (*r* = −0.190, *p* < 0.001), SSRS (*r* = −0.107, *p* < 0.001), and CQ-11D (*r* = −0.370, *p* < 0.001); BC demonstrated significant positively correlations with SSRS (*r* = 0.073, *p* < 0.05) and CQ-11D (*r* = 0.422, *p* < 0.001), whereas SSRS showed significantly positively correlated with CQ-11D (*r* = 0.229, *p* < 0.001).

### 3.3 The effect of ACEs and CMQL older adults

We delved into the association between ACEs and CQ-11D scores in older adults. Following univariate analysis, variables demonstrating significant associations were incorporated into a stepwise multiple linear regression model. Linear regression in Table 2 showed that each additional ACE category was associated with a 0.019-point reduction in CQ-11D score (B = −0.019, *P*<0.001), adjusting for age, chronic diseases, education level and sex.

**Table 2:** Results of stepwise linear regression.

Variables	*B*	S. E	*t*	*P*	95%CI	VIF
Constant	1.003	0.025	39.974	<0.001	0.953 ~ 1.052	
Age	−0.027	0.003	−7.935	<0.001	−0.034 ~ −0.020	1.076
Adverse childhood experiences	−0.019	0.003	−6.649	<0.001	−0.024 ~ −0.013	1.089
Chronic diseases	−0.069	0.013	−5.506	<0.001	−0.094 ~ −0.045	1.091
Education level	0.015	0.006	2.622	0.009	0.004 ~ 0.026	1.079
Sex	−0.021	0.009	−2.212	0.027	−0.039 ~ −0.002	1.017

*R*^2^ = 0.170. Urban-Country distribution and nationality were dropped in this model.

### 3.4 The robustness test

**Figure 1:**
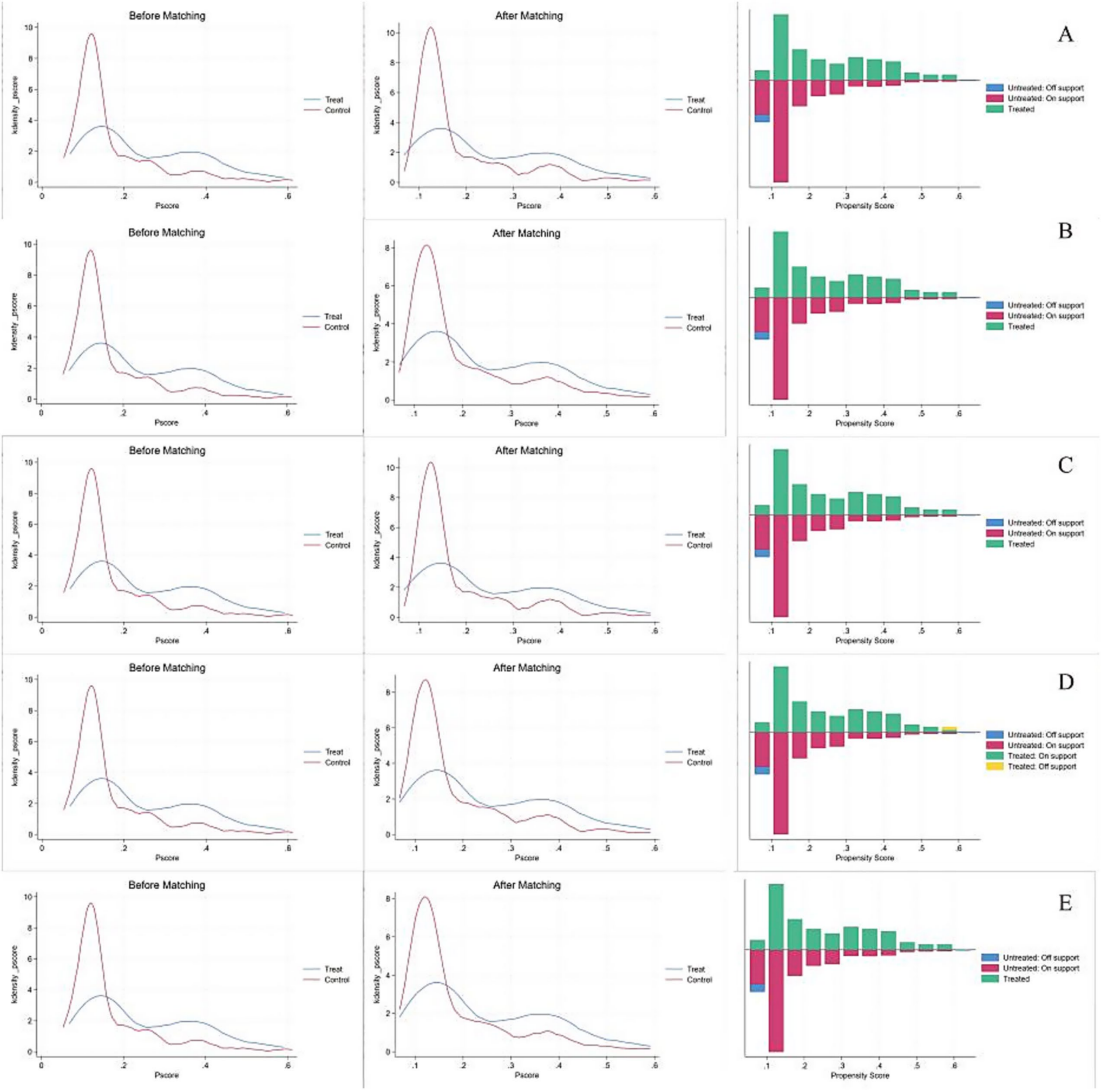
Panels **(A–E)** shows the before and after kernel density plots and balance test plots for the one-to-one matching method, one-to-four matching method, linear regression matching method, Kernel matching method, and Radius matching method, respectively.

The kernel density plots were shown in Figure 1. PSM using five methods yielded consistent results (ATT range: 0.035–0.043, all *p* < 0.001), confirming robustness of the association between ACEs exposure and CQ-11D (Table 3).

**Table 3:** Robustness test based on the matching approach.

Methods		ATT	SE	*t*
k-nearest neighbors matching	One-to-one matching	0.043^***^	0.013	3.30
	One-to-four matching	0.041^***^	0.013	3.21
	Radius matching	0.035^***^	0.012	2.90
Overall matching	Kernel matching	0.037^***^	0.011	3.21
	Local linear regression matching	0.039^***^	0.013	3.03

***p < 0.001.

### 3.5 Heterogeneity test

Differences in the scores of CQ-11D among different sex and urban-country distribution groups were examined in this study. The specific heterogeneity results were presented in Supplementary Table 5. It was observed that there were no discernible differences in the association between ACEs exposure and the scores of CQ-11D among older adults based on sex and urban–rural distribution.

### 3.6 Mechanism analysis

Using IBM SPSS Amos 26.0, we conducted an analysis of the relationship with ACEs as the independent variable, the scores of CQ-11D as the dependent variable, and chronic diseases, BC, and the scores of SSRS as mediating variables. This analysis controlled for statistically significant factors identified in univariate analysis. The structural equation model fit indices were as follows: (RMSEA = 0.033; CFI = 0.928; TLI = 0.907; *χ*^2^/df = 2.238).

**Figure 2:**
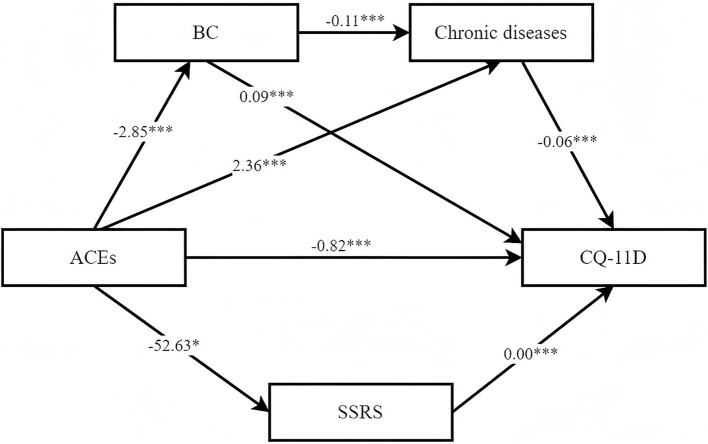
Mediating effect model. ^***^P < 0.001, ^**^P < 0.01, ^*^P < 0.05; BC refers to balance constitution; ACEs refers to adverse childhood experiences; CQ-11D refers to Chinese Medicine Quality of Life Assessment Scale; SSRS refers to social support rating scale.

The structural equation model results were shown in Figure 2, indicating that the indirect effect of ACEs on CMQL through balance constitution, chronic diseases, and social support accounted for 42% of the total effect. The results of the mediation analysis were presented in Table 4, where the 95% confidence intervals for the four mediation paths do not include 0. This finding indicated that the mediating effects of these four variables were all significant, with effect values of −0.169, −0.268, −0.139, and −0.018, respectively. Furthermore, since the direct effects were not equivalent to 0, it suggested that BC, chronic diseases, and scores of SSRS partially mediated the relationship between ACEs and the scores of CQ-11D.

**Table 4:** Mediated effects model path coefficients.

Pathway relationship	Coefficients	SE	Effects	*p-*value	95%CI	
					Lower limit	Upper limit
Total effect	−1.414	0.627		<0.001	−3.074	−0.789
Direct effect						
ACEs → CQ-11D	−0.820	0.211	0.580	<0.001	−1.934	−0.355
Total indirect effect	−0.594	0.233	0.420	<0.001	−1.225	−0.356
ACEs → SSRS → CQ-11D	−0.169	0.077	0.120	<0.001	−0.381	−0.082
ACEs → BC → CQ-11D	−0.268	0.113	0.190	<0.001	−0.554	−0.147
ACEs → chronic diseases → CQ-11D	−0.139	0.062	0.098	<0.001	−0.312	−0.070
ACEs → BC → chronic diseases → CQ-11D	−0.018	0.009	0.013	<0.001	−0.043	−0.008

BC refers to balance constitution; ACEs refers to adverse childhood experiences; CQ-11D refers to Chinese Medicine Quality of Life Assessment Scale; SSRS refers to social support rating scale.

The remaining related path coefficients were detailed in Table 5. The results indicated that the total effect of ACEs on the scores of CQ-11D was significant (*β* = −0.112, *p* < 0.001). Specifically, “parental long-term illness” (*β* = −0.122, *p* < 0.001), “forced school dropout” (*β* = −0.098, *p* = 0.001), and “parents being indifferent to you” (*β* = −0.081, *p* = 0.008) were significantly associated with lower scores of CQ-11D. ACEs was not only linked to a higher prevalence of chronic diseases (*β* = 0.215, *p* < 0.001) but also associated with lower scores of BC (*β* = −0.118, *p* < 0.001) and SSRS (*β* = −0.063, *p* < 0.05). Additionally, the scores of SSRS exhibited a statistically significant direct effect on the scores of CQ-11D (*β* = 0.186, *p* < 0.001). Similarly, higher scores of BC (*β* = 0.326, *p* < 0.001) were associated with higher scores of CQ-11D, while a greater number of chronic diseases (*β* = −0.200, *p* < 0.001) were associated with lower scores of CQ-11D.

**Table 5:** Mediation effect estimates.

Outcome variables	Predictor variables	Overall fit indices			Regression coefficient		*P*-value
		*R*	*R* ^2^	*F*	*β*(95%*CI*)	*t*	
CQ-11D	ACEs	0.318	0.101	13.882	−0.112(−0.075 ~ −0.024)	−3.815	<0.001
	Parental death	0.308	0.095	12.928	−0.033(−0.041 ~ 0.012)	−1.095	0.274
	Parental physical/mental disability	0.305	0.093	12.655	0.005(−0.038 ~ 0.045)	0.153	0.878
	Parental long-term illness	0.331	0.110	15.179	−0.122(−0.101 ~ −0.034)	−3.972	<0.001
	Poor parental relationships	0.315	0.099	13.605	−0.038(−0.048 ~ 0.012)	−1.184	0.237
	Hunger	0.305	0.093	12.683	−0.017(−0.027 ~ 0.015)	−0.555	0.579
	Poor parental mental health	0.321	0.103	14.117	−0.035(−0.049 ~ 0.015)	−1.052	0.293
	Parents lacking formal education	0.300	0.090	12.167	−0.018(−0.033 ~ 0.018)	−0.605	0.545
	Frequent abuse	0.315	0.099	13.601	−0.049(−0.090 ~ 0.008)	−1.638	0.102
	Forced school dropout	0.328	0.108	14.878	−0.098(−0.056 ~ −0.013)	−3.188	0.001
	Parents being indifferent to you	0.322	0.104	14.252	−0.081(−0.071 ~ −0.011)	−2.672	0.008
	Parental divorce	0.304	0.092	12.512	−0.010(−0.057 ~ 0.040)	−0.328	0.743
	Others	0.299	0.089	12.107	0.032(−0.023 ~ 0.083)	1.099	0.272
	Chronic diseases	0.357	0.127	17.956	−0.200(−0.113 ~ −0.063)	−6.924	<0.001
	BC	0.435	0.189	28.743	0.326(0.094 ~ 0.132)	11.676	<0.001
	SSRS	0.349	0.121	17.039	0.186(0.003 ~ 0.005)	6.358	<0.001
BC	ACEs	0.273	0.075	9.936	−0.118(−0.225 ~ −0.076)	−3.967	<0.001
	Parental death	0.261	0.068	9.005	−0.039(−0.128 ~ 0.027)	−1.289	0.198
	Parental physical/mental disability	0.260	0.067	8.917	−0.038(−0.198 ~ 0.047)	−1.215	0.225
	Parental long-term illness	0.261	0.068	9.003	−0.043(−0.166 ~ 0.031)	−1.353	0.176
	Poor parental relationships	0.255	0.065	8.566	−0.007(−0.099 ~ 0.080)	−0.206	0.837
	Hunger	0.281	0.079	10.597	−0.098(−0.162 ~ −0.036)	−3.080	0.002
	Poor parental mental health	0.262	0.069	9.078	−0.032(−0.139 ~ 0.049)	−0.936	0.350
	Parents lacking formal education	0.250	0.062	8.211	0.031(−0.036 ~ 0.115)	1.023	0.306
	Frequent abuse	0.254	0.064	8.478	−0.008(−0.166 ~ 0.125)	−0.276	0.782
	Forced school dropout	0.276	0.076	10.146	−0.086(−0.152 ~ −0.025)	−2.725	0.007
	Parents being indifferent to you	0.252	0.064	8.369	−0.001(−0.091 ~ 0.087)	−0.044	0.965
	Parental divorce	0.261	0.068	9.042	−0.055(−0.279 ~ 0.008)	−1.847	0.065
	others	0.253	0.064	8.452	−0.016(−0.199 ~ 0.115)	−0.531	0.596
	Chronic diseases	0.289	0.083	11.198	−0.152(−0.268 ~ −0.120)	−5.138	<0.001
Chronic diseases	ACEs	0.317	0.101	13.769	0.215(0.157 ~ 0.272)	7.329	<0.001
	Parental death	0.255	0.065	8.540	0.055(−0.005 ~ 0.116)	1.813	0.070
	Parental physical/mental disability	0.246	0.060	7.916	0.015(−0.072 ~ 0.119)	0.487	0.626
	Parental long-term illness	0.254	0.065	8.508	0.054(−0.009 ~ 0.144)	1.724	0.085
	Poor parental relationships	0.250	0.063	8.230	0.027(−0.040 ~ 0.099)	0.835	0.404
	Hunger	0.288	0.083	11.165	0.138(0.060 ~ 0.158)	4.362	<0.001
	Poor parental mental health	0.252	0.063	8.350	0.020(−0.051 ~ 0.095)	0.587	0.557
	Parents lacking formal education	0.239	0.057	7.447	−0.007(−0.066 ~ 0.052)	−0.234	0.815
	Frequent abuse	0.248	0.062	8.090	0.027(−0.063 ~ 0.164)	0.880	0.379
	Forced school dropout	0.277	0.076	10.203	0.094(0.026 ~ 0.126)	3.010	0.003
	Parents being indifferent to you	0.249	0.062	8.124	0.039(−0.024 ~ 0.114)	1.270	0.204
	Parental divorce	0.240	0.058	7.546	−0.005(−0.121 ~ 0.103)	−0.163	0.870
	Others	0.241	0.058	7.567	−0.072(−0.275 ~ −0.030)	−2.447	0.015
SSRS	ACEs	0.234	0.055	8.001	−0.063(−2.446 ~ −0.096)	−2.122	0.034
	Parental death	0.273	0.075	9.945	−0.049(−2.225 ~ 0.218)	−1.612	0.107
	Parental physical/mental disability	0.270	0.073	9.723	−0.011(−2.283 ~ 1.580)	−0.357	0.721
	Parental long-term illness	0.268	0.072	9.525	0.005(−1.423 ~ 1.684)	0.165	0.869
	Poor parental relationships	0.289	0.084	11.232	−0.090(−3.386 ~ −0.562)	−2.744	0.006
	Hunger	0.267	0.071	9.475	0.037(−0.396 ~ 1.591)	1.180	0.238
	Poor parental mental health	0.272	0.074	9.861	0.051(−0.355 ~ 2.618)	1.494	0.136
	Parents lacking formal education	0.267	0.071	9.483	−0.017(−1.524 ~ 0.856)	−0.551	0.582
	Frequent abuse	0.297	0.088	11.954	−0.101(−6.161 ~ −1.567)	−3.301	0.001
	Forced school dropout	0.268	0.072	9.545	0.005(−0.920 ~ 1.088)	0.164	0.870
	Parents being indifferent to you	0.285	0.081	10.909	−0.068(−2.957 ~ −0.159)	−2.184	0.029
	Parental divorce	0.290	0.084	11.337	−0.087(−5.657 ~ −1.122)	−2.933	0.003
	Others	0.270	0.073	9.670	−0.016(−3.150 ~ 1.806)	−0.532	0.595

*p < 0.01; −for duplicate data, do not repeat; gender, domicile, residence, population type, nationality, education level, childhood health status and family history of chronic diseases as control variables.

BC refers to balance constitution; ACEs refers to adverse childhood experiences; CQ-11D refers to Chinese Medicine Quality of Life Assessment Scale; SSRS refers to social support rating scale.

## 4 Discussion

This study aimed to examine the association between ACEs and CMQL among older adults, while exploring the mediating role of BC, chronic diseases, and perceived social support. The findings revealed that ACEs was associated with CMQL, and that ACEs indirectly affected CMQL by affecting BC, chronic diseases and perceived social support. This suggested that these three mediators played a key role in explaining the relationship between ACEs and CMQL in older adults individuals.

The CQ-11D scores among older adults in Sichuan Province were found to be 0.91 (0.79, 0.95), indicating a relatively suboptimal quality of life with potential for enhancement in the future. Notably, there was a significant disparity in scores between sex, with a score of 0.92 (0.81, 0.96) for males and 0.88 (0.77, 0.94) for females. These scores were by Zhu and Pan in mainland China. lower than the findings of previous studies by Zhu and Pan in mainland China (12, 25). This variance could be attributed to the broader range of study subjects and regions in the previous research, as well as the relatively smaller sample size of older adults individuals included. Consequently, this study sheds light on the inadequate quality of life among the older adults population, identifying key factors that contribute to this issue. It lays a foundation for implementing measures aimed at enhancing and improving the quality of life for older adults. This finding underscores the imperative for relevant authorities to implement targeted care and support policies specifically tailored to the female demographic, with the aim of improving the quality of life for older adults in their later years.

In our study, 81.68% of the older adults individuals surveyed reported experiencing ACEs, indicating a significant prevalence of ACEs among the older adults population in Sichuan Province. This percentage is slightly lower than the findings from a study conducted on 3,368 older adults individuals in China between 2013 and 2014, which revealed that 84.46% of older adults individuals had mothers who did not received formal education during their childhood (31). This variance could be attributed to a higher proportion of urban residents in our study, potential exposure to greater educational opportunities and resources during childhood compared to rural areas, potentially resulting in to relatively lower rates of ACEs. In the extensive rural regions of China, older adults individuals are more susceptible to experiences of poverty, hunger, and limited access to education during childhood due to various historical, economic, and cultural factors. Additionally, their levels of educational attainment are typically lower. Therefore, it is essential for the government and society to prioritize the well-being of older adults individuals in rural areas by increasing investments in education, enhancing social security systems, improving the quality of healthcare services available in rural settings.

In our study, older adults individuals with a higher number of ACEs tended to exhibit lower levels of CMQL. Specifically, within the categories of ACEs, we identified that experiences such as “Parental long-term illness,” “Forced school dropout,” and “Parents being indifferent to you” were significantly associated to diminished quality of life.

To begin, “Parental long-term illness” was notably associated with the CMQL among older adults individuals. Previous studies have shown that socio-economic status experienced during childhood has a significant positive impact on health-related quality of life in middle-older adults and older adults populations, indicating that individuals raised in families with higher socio-economic status during childhood often enjoy better health-related quality of life in later stages of life (32). According to life course theory, the impacts of early life experiences are cumulative, with prolonged exposure to ACEs correlating with poorer health outcomes (31). The socio-economic status of parents during an individual’s upbringing typically has a lasting effect. Therefore, enduring parental illness, likely linked to relatively lower socio-economic status, could have a negative influence on their children’s well-being.

Secondly, “Forced school dropout” was significantly associated with the decline of CMQL among older adults individuals. Schools, as structured and institutionalized settings, play a crucial role in the socialization process of children. Learning to engage and communicate with peers, developing the capacity to understand different perspectives, and acquiring skills to function effectively in society are essential aspects of school environment (33). Children who are compelled to drop out of school in their formative years often lack or have limited exposure to these crucial aspects of school life, leading to a deficit in social interaction and communication with peers.

The disruption of their essential socialization process significantly heightens the risk of poor social adaptability and emotional disorders in adulthood. Consequently, the negative effects of early school withdrawal accumulate over time, leading to a detrimental impact on the well-being of older adults in later life. This observation aligns with previous studies indicating that social isolation during childhood can substantially diminish the quality of life in later years (32). Moreover, discontinuing education can contribute to lower educational achievements, potentially leading to declines in cognitive abilities and an increased susceptibility to cognitive impairment in older age (34).

Lastly, “Parents being indifferent to you” was also found to be correlated with the CMQL. Enduring emotional neglect from family members during childhood significantly heightens the likelihood of depression among older adults individuals (35). Depression, characterized by negative emotional experiences, can profoundly impact the quality of life in adulthood and later in old age, increasing the susceptibility to chronic diseases. The adverse impacts of emotional neglect during childhood accumulate over time, ultimately impacting the happiness and quality of life in old age. This observation is in line with previous research indicating childhood social isolation substantially diminishes the quality of life in later years. This findings underscore the indispensable role that the family dynamics play in the formative years of an individual. The objective environment and the quality of intimate relationships within the family directly shape the development of children’s personality and have lasting effects on their long-term quality of life. In managing family life, parents should be attentive to meeting both the material and emotional needs of their children. It is crucial to avoid any form of direct or indirect physical and psychological abuse to ensure the well-being and healthy development of children.

The BC and chronic diseases both act as simple mediators in the relationship between ACEs and CMQL in older adults. This study demonstrated that older adults individuals who have encountered ACEs are less likely to exhibit a balanced traditional Chinese constitution and are more susceptible to chronic diseases, a finding that aligns prior studies (36). The potential factors contributing to this pattern include experiences of childhood hunger, deprivation, and physical or psychological abuse by others.

Furthermore, possessing an imbalanced traditional Chinese constitution and dealing with chronic diseases were both associated with lower level of CMQL among older adults individuals. BC and chronic diseases functioned as a sequential mediating mechanism bridging the gap between childhood adversity and the quality of life of older adults individuals. The study also indicates that older adults individuals with imbalanced traditional Chinese constitutions are at a heightened risk of developing chronic diseases. For this demographic, the adverse effects of ACEs may not dissipate over time but instead persist, impacting their quality of life at a profound level. These early-life experiences subtly shape their TCMC, rendering them more predisposed to specific chronic diseases in old age, consequently markedly diminishing their quality of life (37). This highlights the indispensable role of individual agency in later stages of life. Older adults individuals who have faced ACEs can potentially reduce the occurrence of chronic diseases through positive lifestyle modifications and targeted clinical interventions addressing TCMC. By doing so, they can enhance their sense of well-being and elevate their and quality of life in old age.

In our study, the inverse relationship between the ACEs and the scores of SSRS suggests that individuals with higher ACEs may perceive lower levels of social support, which serves as a protective factor for CMQL of older adults. Specifically, older adults individuals with greater social support tend to exhibit higher levels of CMQL, a trend consistent with findings from previous studies (38, 39). When confronted with challenges, receiving increased social support makes older adults individuals more inclined to adopt a positive attitude toward life, fostering feelings of joy, heightened self-assurance, and other positive emotional outcomes. This emphasizes the importance of interventions focused on strengthening social support networks for individuals who have undergone ACEs, as these networks play a crucial role in promoting overall well-being and resilience. By operating on both physiological and psychological levels, bolstering social support networks significantly enhance CMQL.

Perceived social support can significantly impact the management of chronic diseases, influencing psychological resilience and self-management behaviors (40). Stronger perceived social support is usually associated with healthier behaviors and adherence to treatment plans. This support network can offer emotional comfort, practical assistance, and valuable information that contribute to improved health outcomes. Conversely, individuals lacking adequate social support may experience heightened stress levels, feelings of social isolation, and ultimately encounter poorer health outcomes. The absence of a robust support system can hinder one’s ability to cope effectively with the challenges of chronic diseases, potentially leading to increased health complications and a decreased quality of life (41). In alignment with the TCMC theory, which emphasizes that only a balanced constitution signifies good health, BC emerges as a critical factor in resilience against illnesses and overall well-being (42). Individuals with a balanced constitution may be better equipped to leverage the benefits of social support, enhancing their ability to manage chronic diseases effectively and maintain a higher quality of life.

However, this study still had some limitations. Firstly, the cross-sectional study, based on self-assessment data from Sichuan Province, may be prone to individual subjective biases, potentially affecting the study’s objectivity and reliability. Future studies endeavors could broaden the scope of data collection to encompass older adults populations from diverse geographical regions. This expansion would serve to mitigate individual biases and enhance the generalizability of the findings. Secondly, as a cross-sectional study, it cannot establish causal relationships. Subsequent studies could opt for longitudinal or cohort designs, enabling the tracking of changes over time, identification of developmental trends, and comparison of outcomes across different demographic groups. This approach would facilitate a more comprehensive analysis of the study variables. Thirdly, the questionnaire used to assess ACEs was self-constructed. Future improvements could involve revisions that incorporate more rigorous assessments of reliability. Lastly, due to constraints, not all potential covariate variables were included in this study. Consequently, it is impossible to completely rule out all potential impacts.

This study indicated that relatively low level of CMQL among older adults in Sichuan Province. ACEs were associated with lower level of CMQL of older adults. Furthermore, perceived social support and BC were found to play significant mediating roles in enhancing CMQL, whereas chronic diseases exert a detrimental effect. It is important to acknowledge the limitations of inferring causal relationships from cross-sectional data. These findings underscored the importance of addressing ACEs and their enduring consequences, as well as promoting perceived social support and TCM-based health interventions to improve the quality of life among older adults.

## Data Availability

The raw data supporting the conclusions of this article will be made available by the authors, without undue reservation.

## References

[1] Liu H Yao Y Yang Y Wang J Yu J Xu X. Chronic lung diseases and depressive symptoms in older adults: insights from observational studies and Mendelian randomization. J Multidiscip Healthc. (2025) 18:3465–75. doi: 10.2147/JMDH.S515745, PMID: 40538380 PMC12178258

[2] Jiang M Yao Y Xia X Kong Y Zhang N. The impact of perceived Community Services for the Elderly on self-rated health: an analysis utilizing a mediated latent growth model. J Multidiscip Healthc. (2024) 17:4383–96. doi: 10.2147/JMDH.S476502, PMID: 39267893 PMC11390836

[3] Zhang N Yao Y Li L Sun M Zhou B Fu H. Deprivation-related adverse childhood experiences and cognitive function among older adults: mediating role of depression symptoms. Child Abuse Negl. (2024) 158:107088. doi: 10.1016/j.chiabu.2024.107088, PMID: 39406057

[4] Song J Liu L Miao H Xia Y Li D Yang J. Urban health advantage and penalty in aging populations: a comparative study across major megacities in China. Lancet Reg Health West Pac. (2024) 48:101112. doi: 10.1016/j.lanwpc.2024.101112, PMID: 38978965 PMC11228801

[5] Haraldstad K Wahl A Andenæs R Andersen JR Andersen MH Beisland E. A systematic review of quality of life research in medicine and health sciences. Qual Life Res. (2019) 28:2641–50. doi: 10.1007/s11136-019-02214-9, PMID: 31187410 PMC6761255

[6] Xie S Wang D Wu J Liu C Jiang W. Comparison of the measurement properties of SF-6Dv2 and EQ-5D-5L in a Chinese population health survey. Health Qual Life Outcomes. (2022) 20:96. doi: 10.1186/s12955-022-02003-y, PMID: 35710429 PMC9202323

[7] Li DL Wang ZT Nie XY Luo N Wu YB Pan CW. EQ-5D-5L population norms for China derived from a National Health Survey. Value Health. (2024) 27:1108–20. doi: 10.1016/j.jval.2024.04.014, PMID: 38677363

[8] Yao Q Yang F Zhang X Qi J Li H Wu Y. EQ-5D-5L population scores in mainland China: results from a nationally representative survey 2021. Value Health. (2024) 27:1573–84. doi: 10.1016/j.jval.2024.06.012, PMID: 38977191

[9] Mao Z Ahmed S Graham C Kind P Sun YN Yu CH. Similarities and differences in health-related quality-of-life concepts between the east and the west: a qualitative analysis of the content of health-related quality-of-life measures. Value Health Reg Issues. (2021) 24:96–106. doi: 10.1016/j.vhri.2020.11.007, PMID: 33524902

[10] Mao Z Ahmed S Graham C Kind P. Exploring subjective constructions of health in China: a Q-methodological investigation. Health Qual Life Outcomes. (2020) 18:165. doi: 10.1186/s12955-020-01414-z, PMID: 32493342 PMC7268713

[11] Zhou J Xu L Pan J Wang M Zhou P Wang W. A comparative study of Chinese medicine quality of life assessment scale (CQ-11D) and EQ-5D-5L and SF-6D scales based on Chinese population. Qual Life Res. (2024) 33:113–22. doi: 10.1007/s11136-023-03512-z, PMID: 37695478 PMC10784339

[12] Zhu W Zhang M Pan J Shi L Gao H Xie S. Valuing Chinese medicine quality of life-11 dimensions (CQ-11D) health states using a discrete choice experiment with survival duration (DCETTO). Health Qual Life Outcomes. (2023) 21:99. doi: 10.1186/s12955-023-02180-4, PMID: 37612664 PMC10463386

[13] Liang M Fu X Gao P Zhu W. Comparative analysis on euro QOL-5 dimensions and short form 6D in quality of life scale. Chin Health Econ. (2014) 33:9–11.

[14] Zhu YB. Measurement and evaluation of QOL. Beijing: People’s Military Surgeon Publishing House (2010).

[15] Lin L Wang HH Lu C Chen W Guo VY. Adverse childhood experiences and subsequent chronic diseases among middle-aged or older adults in China and associations with demographic and socioeconomic characteristics. JAMA Netw Open. (2021) 4:e2130143. doi: 10.1001/jamanetworkopen.2021.30143, PMID: 34694390 PMC8546496

[16] Wagner C Carmeli C Jackisch J Kivimäki M van der Linden BWA Cullati S. Life course epidemiology and public health. Lancet Public Health. (2024) 9:e261–9. doi: 10.1016/S2468-2667(24)00018-5, PMID: 38553145

[17] Lin L Cao B Chen W Li J Zhang Y Guo VY. Association of Adverse Childhood Experiences and Social Isolation with Later-Life Cognitive Function among Adults in China. JAMA Netw Open. (2022) 5:e2241714. doi: 10.1001/jamanetworkopen.2022.41714, PMID: 36367722 PMC9652754

[18] Tabatabaei FS Delbari A Bidkhori M Saatchi M Zanjari N Hooshmand E. The role of childhood circumstances on social conditions and health of middle-aged and older adults: Ardakan cohort study on aging (ACSA). J Appl Gerontol. (2024) 43:577–87. doi: 10.1177/07334648231213731, PMID: 38018420

[19] Dong Y Cheng L Cao H. Impact of informal social support on the mental health of older adults. Front Public Health. (2024) 12:1446246. doi: 10.3389/fpubh.2024.1446246, PMID: 39391160 PMC11464432

[20] Lindsay Smith G Banting L Eime R O’Sullivan G van Uffelen JGZ. The association between social support and physical activity in older adults: a systematic review. Int J Behav Nutr Phys Act. (2017) 14:56. doi: 10.1186/s12966-017-0509-8, PMID: 28449673 PMC5408452

[21] Ahmed W Muhammad T Muneera K. Prevalence of early and late onset of chronic diseases and multimorbidity and its association with physical, mental and functional health among older Indian adults. BMC Geriatr. (2023) 23:563. doi: 10.1186/s12877-023-04264-8, PMID: 37710170 PMC10502995

[22] Yang CC Yen SJ Chiu XD Wu KC Ye SC Su SH. Decision tree-based body constitution diagnosis system for traditional Chinese medicine. Evid Based Complement Alternat Med. (2022) 2022:1–10. doi: 10.1155/2022/5560087, PMID: 35295930 PMC8920620

[23] Sun Z Ping P Li Y Feng L Liu F Zhao Y. Relationships between traditional Chinese medicine constitution and age-related cognitive decline in Chinese centenarians. Front Aging Neurosci. (2022) 14:870442. doi: 10.3389/fnagi.2022.870442, PMID: 35615593 PMC9126494

[24] Liang X Wang Q Jiang Z Li Z Zhang M Yang P. Clinical research linking traditional Chinese medicine constitution types with diseases: a literature review of 1639 observational studies. J Tradit Chin Med. (2020) 40:690–702. doi: 10.19852/j.cnki.jtcm.2020.04.019, PMID: 32744037

[25] Pan J Han Q Zhou P Zhou J Zhang M Zhu W. Assessing health-related quality of life of Chinese population using CQ-11D. Health Qual Life Outcomes. (2024) 22:34. doi: 10.1186/s12955-024-02250-1, PMID: 38637793 PMC11027529

[26] Qu X Xiong HZ Qu DQ Liu H Xu XX Sun R. Correlation analysis of traditional Chinese medicine constitution and metabolic indexes in general physical examination people. Endocr Metab Immune Disord Drug Targets. (2025) 25:560–8. doi: 10.2174/0118715303302433240918104124, PMID: 39364874

[27] Yang L. Childhood adversity and trajectory of chronic diseases in later life. J Soc Dev. (2023) 10:62–82.

[28] Li Y Lu J. Study of the effect of childhood adversity on depression among Chinese older adults. Popul J. (2020) 42:56–69. doi: 10.16405/j.cnki.1004-129X.2020.04.005

[29] Liu R Li J. Effects of adverse childhood experiences on health among middle-aged and older adults in the perspective of addressing mortality selection. J Yunnan Minzu Univ. (2022) 39:66–75. doi: 10.13727/j.cnki.53-1191/c.20221104.005

[30] Liu L Gou Z Zuo J. Social support mediates loneliness and depression in elderly people. J Health Psychol. (2016) 21:750–8. doi: 10.1177/1359105314536941, PMID: 24925547

[31] Liu Y Dai W Yang Y Ning X Huang Y Luo Y. Adverse childhood experiences and multimorbidity among middle-aged and older adults: evidence from China. Child Abuse Negl. (2024) 158:107100. doi: 10.1016/j.chiabu.2024.107100, PMID: 39514998

[32] Beck A Franz CE Xian H Vuoksimaa E Tu X Reynolds CA. Mediators of the effect of childhood socioeconomic status on late midlife cognitive abilities: a four decade longitudinal study. Innov Aging. (2018) 2:igy 003. doi: 10.1093/geroni/igy003, PMID: 30465026 PMC6176967

[33] Wentzel KR Looney L. Socialization in school settings. In: Grusec JE Hastings PD, editors. Handbook of socialization: theory and research. (New York: Guilford Press) (2007). 382–403.

[34] Ma Y Xiang Q Yan C Liao H Wang J. Relationship between chronic diseases and depression: the mediating effect of pain. BMC Psychiatry. (2021) 21:436. doi: 10.1186/s12888-021-03428-3, PMID: 34488696 PMC8419946

[35] Xiang X Wang X. Childhood adversity and major depression in later life: a competing-risks regression analysis. Int J Geriatr Psychiatry. (2021) 36:215–23. doi: 10.1002/gps.5417, PMID: 32869351

[36] Yin H Qiu X Zhu Y Yang Q. Adverse childhood experiences affect the health of middle-aged and older people in China: the multiple mediating roles of sleep duration and life satisfaction. Front Psych. (2023) 14:1092971. doi: 10.3389/fpsyt.2023.1092971, PMID: 37032944 PMC10073436

[37] Haczkewicz KM Shahid S Finnegan HA Monnin C Cameron CD Gallant NL. Adverse childhood experiences (ACEs), resilience, and outcomes in older adulthood: a scoping review. Child Abuse Negl (2024) 106864. doi: 10.1016/j.chiabu.2024.106864, PMID: 38926006

[38] Shen T Li D Hu Z Li J Wei X. The impact of social support on the quality of life among older adults in China: an empirical study based on the 2020 CFPS. Front Public Health. (2022) 10:914707. doi: 10.3389/fpubh.2022.914707, PMID: 36159275 PMC9490036

[39] Yang C Jia C Yin S Mao Z Cui D. The effect of informal social support on the health of Chinese older adults: a cross-sectional study. BMC Public Health. (2023) 23:1017. doi: 10.1186/s12889-023-15837-y, PMID: 37254142 PMC10227956

[40] Lin C Zhu X Wang X Wang L Wu Y Hu X. The impact of perceived social support on chronic disease self-management among older inpatients in China: the chain-mediating roles of psychological resilience and health empowerment. BMC Geriatr. (2025) 25:284. doi: 10.1186/s12877-025-05902-z, PMID: 40287628 PMC12032646

[41] Reblin M Uchino BN. Social and emotional support and its implication for health. Curr Opin Psychiatry. (2008) 21:201–5. doi: 10.1097/YCO.0b013e3282f3ad89, PMID: 18332671 PMC2729718

[42] Hsu MF Tang PL Pan TC Hsueh KC. Different traditional Chinese medicine constitution is associated with dietary and lifestyle behaviors among adults in Taiwan. Medicine. (2022) 101:e30692. doi: 10.1097/MD.0000000000030692, PMID: 36181077 PMC9524912

