# Supplementary material for: Exploring the mediating factors linking adverse childhood experiences to traditional Chinese quality of life among older adults: a multi-factorial analysis
Source: Front Public Health. 2025 Sep 16;13:1598440. doi: 10.3389/fpubh.2025.1598440 (PMC12481901; doi:10.3389/fpubh.2025.1598440)
Supplement: Supplementary file 1 [file Supplementary_file_1.docx]

| **Supplementary Table 1** Applicability Test. | | |
| --- | --- | --- |
| KMO Quantity of Sample Suitability | | 0.931 |
| Bartlett’s spherical test | Approximate chi-square | 14723.896 |
|  | Degrees of freedom | 253 |
|  | Significance | ＜0.001 |

| **Supplementary Table 2** Principal Component Analysis. | | | | | | | | | |
| --- | --- | --- | --- | --- | --- | --- | --- | --- | --- |
| Component | Initial EIGENVALUE | | | Extract the Sum of the Squares of the Loads | | | Rotational Load Sum of Squares | | |
|  | Total | % of Variance | Cumulative % | Total | % of Variance | Cumulative % | Total | % of Variance | Cumulative % |
| 1 | 9.265 | 40.284 | 40.284 | 9.265 | 40.284 | 40.284 | 5.884 | 25.584 | 25.584 |
| 2 | 2.199 | 9.562 | 49.846 | 2.199 | 9.562 | 49.846 | 3.279 | 14.255 | 39.839 |
| 3 | 1.553 | 6.752 | 56.597 | 1.553 | 6.752 | 56.597 | 3.148 | 13.688 | 53.527 |
| 4 | 1.156 | 5.026 | 61.623 | 1.156 | 5.026 | 61.623 | 1.862 | 8.096 | 61.623 |

| **Supplementary Table 3** Results of Convergent Validity and Combined Reliability Tests for Each Dimension of the Scale. | | | | |
| --- | --- | --- | --- | --- |
| Pathway Relationship | 载荷 | AVE | CR | Cronbach’s α |
| BC→TCMCC | 0.713 | 0.578 | 0.925 | 0.821 |
| DHC→TCMCC | 0.835 |  |  |  |
| PDC→TCMCC | 0.828 |  |  |  |
| QDC→TCMCC | 0.778 |  |  |  |
| ISC→TCMCC | 0.767 |  |  |  |
| QSC→TCMCC | 0.762 |  |  |  |
| BSC→TCMCC | 0.760 |  |  |  |
| YIDC→TCMCC | 0.706 |  |  |  |
| YADC→TCMCC | 0.679 |  |  |  |
| subjective support→SSRS | 0.785 | 0.585 | 0.809 | 0.831 |
| objective support→SSRS | 0.785 |  |  |  |
| the utilization of social support→SSRS | 0.723 |  |  |  |
| JL→CQ-11D | 0.762 | 0.414 | 0.884 | 0.861 |
| FZ→CQ-11D | 0.719 |  |  |  |
| PL→CQ-11D | 0.649 |  |  |  |
| XH→CQ-11D | 0.558 |  |  |  |
| TY→CQ-11D | 0.522 |  |  |  |
| TT→CQ-11D | 0.482 |  |  |  |
| DB→CQ-11D | 0.737 |  |  |  |
| SY→CQ-11D | 0.730 |  |  |  |
| JS→CQ-11D | 0.665 |  |  |  |
| SM→CQ-11D | 0.623 |  |  |  |
| XD→CQ-11D | 0.563 |  |  |  |

| **Supplementary Table 4** Correlation Analysis. | | | | | |
| --- | --- | --- | --- | --- | --- |
| Variables | Adverse childhood experiences | Chronic diseases | Balance constitution | SSRS | CQ-11D |
| Adverse childhood experiences | 1 | — | — | — | — |
| Chronic diseases | 0.248**^***^** | 1 | — | — | — |
| Balance constitution | -0.138**^***^** | -0.190**^***^** | 1 | — | — |
| SSRS | -0.086**^**^** | -0.107**^***^** | 0.073**^*^** | 1 | — |
| CQ-11D | -0.202**^***^** | -0.370**^***^** | 0.422**^***^** | 0.229**^***^** | 1 |
| ^*^*P*<0.05, ^**^*P*<0.01, ^***^*P*<0.001 | | | | | |

| **Supplementary Table 5** Heterogeneity Analysis Results. | | | | |
| --- | --- | --- | --- | --- |
|  | Sex | | Urban-Country distribution | |
|  | Male | Female | Urban | Country |
| ACEs | 0.0073 | | 0.0019 | |
|  | (0.0132) | | (0.013) | |
| Moderating variables | -0.0222^*^ | | 0.0735^***^ | |
|  | (0.0102) | | (0.0131) | |
| Control variables | All control variables | | All control variables | |
| Interactive items | 0.0676^*^ | | -0.0059 | |
|  | (0.0263) | | 0.0348 | |
| R^2^ | 0.001 | | 0.0028 | |
| ^*^*P*<0.05, ^***^*P*<0.001 | | | | |
